# Pig Face Open Set Recognition and Registration Using a Decoupled Detection System and Dual-Loss Vision Transformer

**DOI:** 10.3390/ani15050691

**Published:** 2025-02-27

**Authors:** Ruihan Ma, Hassan Ali, Malik Muhammad Waqar, Sang Cheol Kim, Hyongsuk Kim

**Affiliations:** 1 Division of Electronics and Information Engineering, Jeonbuk National University, Jeonju 54896, Republic of Korea; maruihan@jbnu.ac.kr (R.M.); 202350496@jbnu.ac.kr (H.A.);; 2 Core Research Institute of Intelligent Robots, Jeonbuk National University, Jeonju 54896, Republic of Korea

**Keywords:** pig face recognition, deep learning, metric learning, open set recognition, registration

## Abstract

Effective pig farming relies on precise and adaptable animal identification methods, particularly in dynamic environments where new pigs are regularly added to the herd. However, pig face recognition is challenging due to high individual similarity, lighting variations, and occlusions. These factors hinder accurate identification and monitoring. To address these issues under Open-Set conditions, we propose a three-phase Pig Face Open-Set Recognition (PFOSR) system. In the Training Phase, we adopt a decoupled design, first training a YOLOv8-based pig face detection model on a small labeled dataset to automatically locate pig faces in raw images. We then refine a Vision Transformer (ViT) recognition model via a dual-loss strategy—combining Sub-center ArcFace and Center Loss—to enhance both inter-class separation and intra-class compactness. Next, in the Known Pig Registration Phase, we utilize the trained detection and recognition modules to extract representative embeddings from 56 identified pigs, storing these feature vectors in a Pig Face Feature Gallery. Finally, in the Unknown and Known Pig Recognition and Registration Phase, newly acquired pig images are processed through the same detection–recognition pipeline, and the resulting embeddings are compared against the gallery via cosine similarity. If the system classifies a pig as unknown, it dynamically assigns a new ID and updates the gallery without disrupting existing entries. Our system demonstrates strong Open-Set recognition, achieving an AUROC of 0.922, OSCR of 0.90, and F1-Open of 0.94. In the closed set, it attains a precision@1 of 0.97, NMI of 0.92, and mean average precision@R of 0.96. These results validate our approach as a scalable, efficient solution for managing dynamic farm environments with high recognition accuracy, even under challenging conditions.

## Simple Summary

Effective management of livestock requires precise identification of individual pigs, which is difficult due to their similar facial features, complex backgrounds, and the necessity to register new pigs as they arrive. Our three-stage system tackles these issues by detecting, recognizing, and dynamically registering new pigs without retaining them. We utilize YOLOv8 for detecting pig faces and employ an enhanced Vision Transformer (ViT) for recognizing those faces. This method allows us to create a feature gallery that can grow as new pigs are added. It ensures accurate and scalable recognition, even in dynamic farming environments, demonstrating strong performance in identifying both known and unknown pigs.

## 1. Introduction

Accurate identification of individual animals is fundamental in modern livestock management, enabling effective monitoring of health, behavior [1], and welfare and optimizing farm productivity [2]. Animal face recognition [3] has emerged as a non-invasive and efficient method for individual identification, playing a crucial role in precision livestock farming. By automating the identification process, farmers can reduce labor costs, improve record-keeping accuracy, and enhance animal welfare. Traditional identification methods, such as ear tags, tattoos, and radio-frequency identification (RFID) devices, have been widely used in the livestock industry [4]. RFID technology, in particular, offers advantages like contactless identification and data storage capabilities. However, it also has notable drawbacks. The initial cost of RFID systems can be high, and the tags may require regular maintenance or replacement due to damage or loss [5]. Additionally, the process of tagging can cause stress and discomfort to the animals, raising concerns about animal welfare. RFID systems can also be susceptible to interference and may not perform reliably in all farm environments.

In recent years, deep learning-based human face recognition technologies [6] have achieved remarkable success, demonstrating high accuracy and robustness under various conditions. These advancements are largely attributed to the ability of deep convolutional neural networks (CNNs) [7] to automatically learn discriminative features from large datasets. Applying deep learning techniques to animal face recognition [8,9,10] holds significant promise, offering the potential to overcome the limitations of traditional methods by providing accurate, efficient, and non-invasive identification. However, pig face recognition presents unique challenges such as non-frontal, extreme head poses, or occluded faces due to grazing or rooting behavior. Due to these challenges, it is hard to recognize pig faces compared to human face recognition.

One significant challenge is the collection of pig face data. Pigs often spend considerable time lying down, making it difficult for cameras to capture clear images of their faces. This behavior necessitates sifting through large volumes of data to extract useful pig face images, which is both time-consuming and labor-intensive [11]. Furthermore, pig face data is affected by several factors that complicate recognition tasks. Pigs have highly similar facial features, leading to high intra-class similarity, which makes it difficult for models to distinguish between individuals. Variations in lighting conditions, complex backgrounds within pig pens, and unrestricted movement lead to significant differences in captured images. The pig’s movements significantly affect the distance between its face and the camera, resulting in noticeable inconsistencies in the size and resolution of pig face images. Pigs often have dirty faces due to their living habits, with mud or debris covering parts of their faces. This introduces occlusions and additional variability, even among images of the same individual, making feature extraction and recognition more challenging.

Compared to cow and sheep face datasets [12,13], pig face datasets are more difficult to collect and process. The behaviors of pigs result in higher variability and noise in the data, necessitating more sophisticated data processing and model training techniques. Pig face detection [14] is also a non-trivial task. Studies have shown that including background in pig face images can negatively impact recognition accuracy because background elements may introduce noise and distract the recognition model from focusing on relevant facial features [15]. Some earlier studies addressed this by manually cropping pig face images to exclude the background [16]. However, manual cropping is impractical for large-scale applications due to the significant labor required.

To overcome these data collection and preprocessing challenges, recent approaches have developed end-to-end pig face detection and recognition systems to automate this process [17]. While these methods have improved efficiency, they still face limitations in dynamic farm environments. Specifically, methods that achieve high recognition accuracy often require retraining and re-labeling when new pigs are introduced. This retraining process increases workload and limits scalability, making such systems less suitable for environments where pig populations frequently change due to the introduction of new piglets and the removal of sick or old pigs. In practical applications, Closed-Set pig recognition systems [18] cannot adapt to these changes without retraining, hindering continuous monitoring and management. Unidentified pigs cannot be tracked or assessed effectively, limiting the utility of such systems in real-world farm operations.

To address these challenges, we focus on the task of pig face Open-Set recognition (PFOSR) [19], which aims to accurately identify known pigs while effectively handling unknown individuals that the model has not encountered during training. PFOSR is essential in dynamic farm environments, ensuring that new pigs can be recognized. A prominent challenge in PFOSR is the high intra-class similarity among pig faces and the absence of unknown individuals during training. Existing methods often rely solely on metric learning loss functions [20], such as ArcFace Loss [21], to enhance inter-class separability by pushing features of different classes apart in the feature space. While effective for distinguishing between classes, relying exclusively on metric learning may not adequately capture intra-class variations, especially when dealing with high variability due to environmental factors and occlusions. Conversely, classification loss functions like Center Loss [22] focus on promoting intra-class compactness by pulling features of the same class together. However, using only classification loss may not sufficiently separate different classes, which is crucial for handling unknown individuals in Open-Set scenarios. To overcome these limitations, we propose a novel three-stage pig face detection, recognition, and registration system designed to operate effectively in dynamic farm environments and address the challenges and complexities involved in PFOSR.

The first stage is the pig face detection and recognition training stage. We build and train the pig face detection and recognition modules in the first phase. A robust pig face detection model is developed to accommodate the variability in pig face appearances under real farm conditions. Using a small, labeled dataset, this detection model learns to accurately identify and localize pig faces in raw images, separating them from cluttered backgrounds. Such a decoupled approach alleviates the need for manual cropping on new, large datasets, as the detection model can be applied to automatically extract pig faces for subsequent steps. Within the same phase, we also train a high-performance pig face recognition model, starting from a modified Vision Transformer (ViT) architecture [23]. The original MLP layer is replaced with a new Embedding layer, and a dual-loss structure—combining Sub-center ArcFace loss [24] and Center Loss—is introduced. By simultaneously increasing inter-class separability and reducing intra-class distance, this strategy enhances the extractor’s discriminative power. The improved model effectively handles subtle differences among pig faces, delivering robust accuracy in both Closed-Set and Open-Set recognition contexts.

The second stage is the Known Pig Registration Phase. After the detection and feature extraction components have been fully trained, the second phase focuses on registering pigs already known to the farm. Known pig images pass through the pig face detection module, and their features are extracted by the trained model. These feature vectors are then stored in a Pig Face Feature Gallery, along with the corresponding pig IDs. This registration mechanism ensures that, for each known pig, representative embeddings exist in the database, reflecting variations in pose, lighting, and other farm conditions. By capturing sufficient diversity in each pig’s features, the gallery becomes a reliable reference for identifying these individuals during subsequent recognition tasks.

The final stage is the Unknown and Known Pig Recognition and Registration Phase. In the final phase, the system manages new incoming images—either from previously registered pigs or from unrecognized pigs. First, the pig face detection module extracts face regions, and the trained feature extractor generates the associated embeddings. These embeddings are then compared against the existing face gallery using a similarity measure (e.g., cosine distance [25]). If a query embedding’s best match exceeds a predefined similarity threshold, the system concludes that the pig is already known, mapping the embedding to an existing ID. Otherwise, the pig is considered unknown: the system creates a new ID and stores its feature vector in the gallery. This dynamic updating mechanism ensures that the pipeline adapts to Open-Set scenarios where new pigs may appear over time, maintaining a relevant and up-to-date gallery of farm animals. By continuously refining its database in this manner, the system can effectively handle both routine farm management tasks and the challenges posed by evolving, large-scale livestock populations.

During inference time, our pig face detection and recognition models automatically detect pig faces and extract their feature vectors. In the Pig Face Feature Matching module, the extracted features are compared with those in the Pig Face Feature Gallery using the distance similarity algorithm. The ID corresponding to the feature with the highest similarity score is assigned to the test sample. This enables accurate recognition and seamless registration of new pigs, enhancing operational efficiency and animal welfare by facilitating accurate and non-invasive monitoring of individual pigs.

Our key contributions are as follows:

- We propose a decoupled pig face detection, recognition, and registration system that reduces manual effort and improves recognition accuracy by focusing on relevant facial features.
- We introduce a dynamic registration mechanism that allows the system to adapt to changes in the pig population without retraining, addressing the Open-Set recognition challenge inherent in PFOSR.
- We design a dual-loss structure that combines metric learning loss and classification loss during training to enhance the discriminative power of the feature extractor. This dual-loss structure captures subtle differences among pig faces while maintaining robustness to intra-class variations, significantly improving recognition accuracy in both Closed-Set and Open-Set scenarios.
- We create a comprehensive pig face dataset comprising a detection dataset, a recognition dataset, a side-face dataset, and a pig face gallery. This dataset facilitates the development of robust detection and recognition models for pig face identification tasks.

By integrating advanced detection and recognition techniques with a dynamic registration mechanism and our proposed dual-loss structure during training, our system effectively addresses the key challenges in pig face Open-Set recognition. This approach not only enhances operational efficiency but also contributes to improved animal welfare by facilitating accurate and non-invasive monitoring of individual pigs.

## 2. Materials and Methods

**Figure 1:**
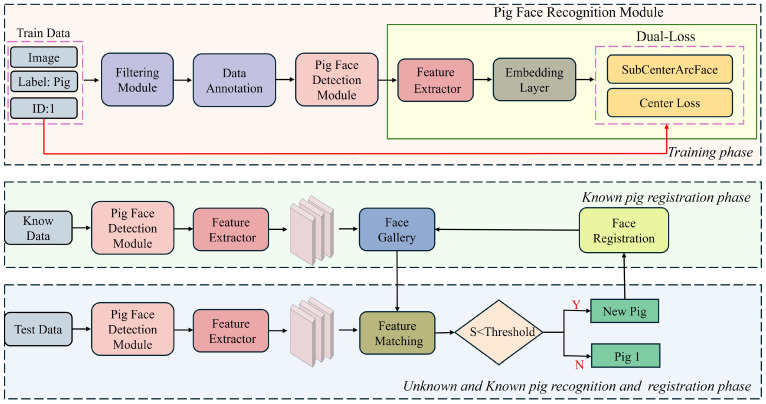
Overview of the proposed three-phase PFOSR pipeline. In the Training Phase, labeled pig images (image + label: pig + ID) are used to develop a robust detection model and a recognition model featuring a dual-loss design (SubCenterArcFace + Center Loss). In the Known Pig Registration Phase, images accompanied by known pig IDs pass through the pig face detection and recognition modules, and the resulting feature embeddings are registered in a Face Gallery. In the Unknown and Known Pig Recognition and Registration Phase, unlabeled images are again processed by the same detection and recognition models; if, for a new embedding, the similarity score with all existing gallery entries falls below a specified threshold, a new pig ID is assigned, and the Face Gallery is updated accordingly. This iterative process enables Open-Set pig face recognition by dynamically integrating newly encountered pigs.

The proposed pig face detection and recognition system is designed to effectively handle dynamic farm environments through Pig Face Open-Set Recognition (PFOSR). The system operates through three primary stages: the known pig face detection and recognition training stage and the second is known pig registration. The third stage comprises unknown pig recognition, registration, and Face Gallery Updation, as illustrated in Figure 1.

### 2.1. System Overview

**Figure 2:**
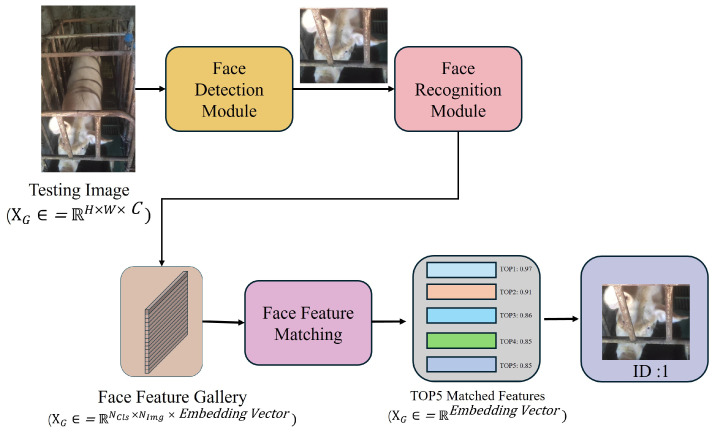
This is the application of our PFOSR system in inference time. During the real-time detection phase of PFOSR, all unknown and known pigs have already been registered. Incoming test images are first processed by the pig face detection and recognition model to extract face features, which are then matched in a 1:N manner against all registered pigs in the feature gallery. The system retrieves the top five matches based on similarity scores and assigns the label of the highest-scoring match to the test image, completing the real-time identification process.

Our proposed Pig Face Open-Set Recognition (PFOSR) system operates in three sequential phases to achieve robust and adaptable pig identification: The training Phase, the Known Pig Registration Phase, and the Unknown and Known Pig Recognition and Registration Phase. In the Training Phase, we build and train high-accuracy pig face detection and recognition models on labeled datasets. The detection model isolates pig faces from cluttered scenes, while the recognition model extracts discriminative features for each identified pig. Once both models are trained, the Known Pig Registration Phase constructs and maintains a feature gallery for already recognized pigs. During this stage, embedding vectors for each known pig are extracted using the trained detection–recognition pipeline and stored in a dynamic repository, allowing new reference data to be incorporated without requiring model retraining. Finally, in the Unknown and Known Pig Recognition and Registration Phase, incoming pig images are passed through the same detection–recognition process and matched against the gallery. If a pig’s features are not found in the gallery, the system automatically assigns a new identity and updates the repository to accommodate the newly encountered animal. This pipeline ensures continuous, real-time pig identification under Open-Set conditions. Figure 2 illustrates the overall architecture. This three-stage pipeline provides a comprehensive solution for pig identification and management, enabling effective livestock monitoring and adapting to changes in the pig population. By adopting this robust method for the advanced detection, recognition, and registration process of pigs, the system lays a reliable foundation for future deployment in farm environments.

### 2.2. Dataset

In this study, we constructed several datasets to train and evaluate our pig face detection and recognition system. These datasets are designed to capture the variability in pig appearances and environmental conditions, ensuring the robustness and generalization ability of our models.

**Figure 3:**
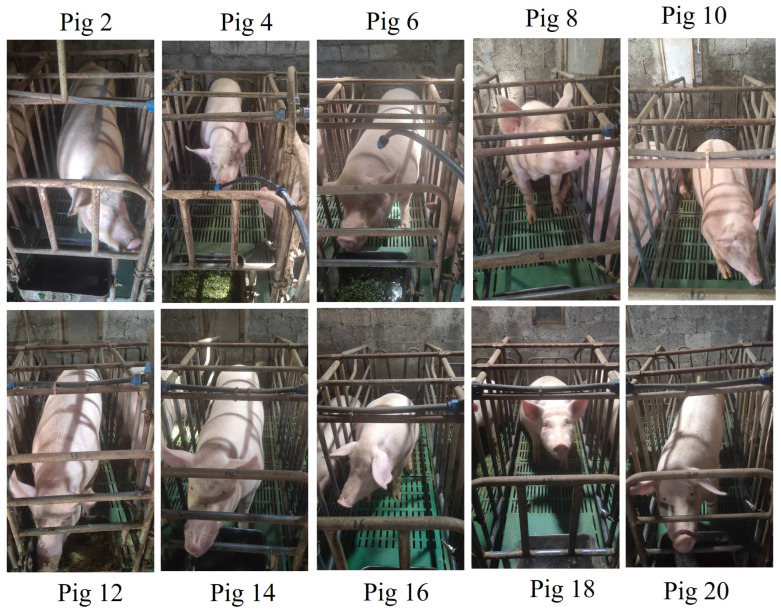
The visualization of the sample images of the Small-Scale Pig Face Detection Dataset.

**Small-Scale Pig Face Detection Dataset.** To enhance the robustness of our pig face detection model and minimize manual annotation efforts for future datasets, we developed a high-quality pig face detection dataset. This dataset comprises 1500 images collected from 20 selected pigs, captured under diverse environmental conditions, lighting, and angles. The images were collected from real farm environments where pigs are housed in enclosures, resulting in some pig faces being partially obscured by bars. During annotation, we included these obstructions within the bounding boxes, as removing them was not feasible. Additionally, the dataset includes various perspectives, such as side and frontal views, adding to its complexity. Each image underwent meticulous manual annotation using the LabelMe tool by trained annotators to ensure precision and consistency. A detailed visualization of the annotation process can be found in the Supplementary Material (Figure S1). The dataset was systematically divided into training, validation, and testing subsets in a 6:2:2 ratio to evaluate the model’s performance comprehensively. The visualization of sample images from this dataset is shown in Figure 3.

**Known Pig Face Recognition Dataset.** This dataset was developed to train a high-performing feature extractor for pig face recognition and to validate the model’s ability to recognize known pig identities. Utilizing the pig face detection model trained on the aforementioned small-scale dataset, we automatically processed a larger collection of images, resulting in 20,000 images from 56 pigs. This approach significantly reduced the need for manual annotation. The dataset is divided into training, validation, and testing subsets with a 6:2:2 ratio, ensuring the model’s robustness in recognizing known pig identities under varied conditions.

**Known Pig Face Feature Dataset.** To build the Pig Face Feature Gallery, we collected an additional 50 high-quality images for each of the 56 known pigs. These images captured under diverse conditions, were processed using our pre-trained pig face detection model to extract pig face regions. Our face feature extractor model then generated feature vectors from these images, which were stored in the gallery to ensure robust feature representation.

**Unknown Pig Face Feature Dataset.** For dynamic registration, we collected a dataset of 9 pigs not included in the training phase, comprising 270 images per pig under varied conditions. From these, 30 images per pig were used for feature registration. These images were processed using our detection model and feature extractor to generate feature vectors, facilitating the seamless integration of new pigs into the feature gallery without retraining.

**Figure 4:**
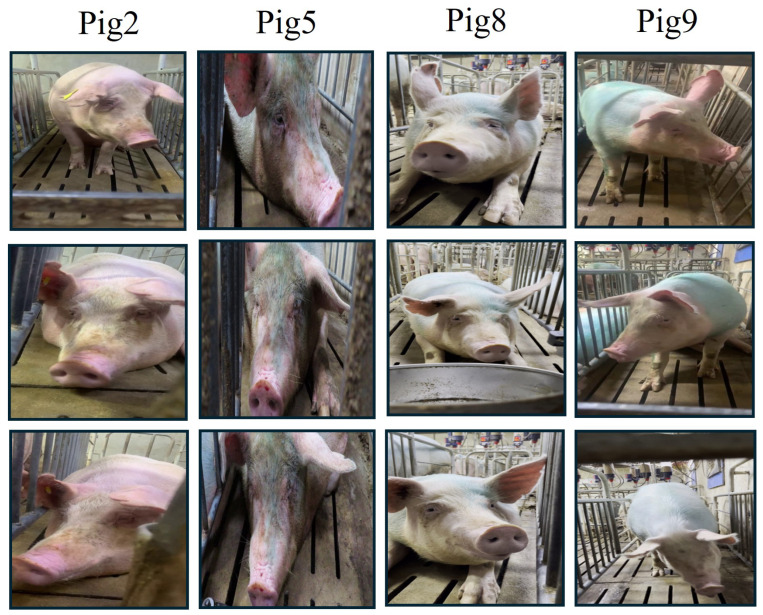
The visualization of the sample images of the Unknown Pig Face Test Dataset.

**Unknown Pig Face Test Dataset.** To evaluate the generalization and adaptability of our recognition model, we collected a test dataset comprising 900 images from the same 9 pigs in the Unknown Pig Face Feature Dataset. These images were captured under diverse conditions to simulate real-world scenarios, including variations in lighting, growth stages, and environmental factors. This dataset was designed to test the model’s ability to recognize previously unseen pigs and assess its robustness and adaptability in dynamic farm environments. Figure 4 provides illustrative samples from the dataset, highlighting representative pig images captured under various lighting and environmental conditions.

**65 Known Pig Face Testing Dataset.** The dataset is designed to evaluate the performance of our pig face recognition model in Closed-Set scenarios after the registration of new classes. This dataset merges both the testing set of the Known Pig Face Recognition Dataset (56 pigs) and the Unknown Pig Face Test Dataset (9 pigs) to simulate a realistic environment where new pigs are introduced and registered into the system.

### 2.3. Training Phase

In this study, we developed a decoupled pig face detection and recognition system comprising several interconnected modules designed for accurate identification of pigs. The system includes the Pig Face Detection Module and the Pig Face Recognition Module.

#### 2.3.1. Pig Face Detection Module

To optimize resource utilization and effectively adapt to both training and testing phases, we developed a high-performance pig face detection model based on the YOLOv8 [26] architecture. By training YOLOv8 on a small, manually annotated dataset of pig faces, the model learned to accurately identify and localize pig faces in various conditions. Once trained, YOLOv8 was utilized to process a larger collection of images, automatically detecting and cropping pig faces without the need for manual intervention. This automation significantly reduced the time and labor associated with dataset creation, ensuring consistency in the extracted face images and enhancing the scalability of our recognition system. This approach allows for efficient processing of extensive datasets, facilitating the development of robust pig face recognition models.

YOLOv8 integrates a CSPDarknet backbone [27] designed to improve the efficiency of feature extraction while maintaining high accuracy. The backbone’s Cross-Stage Partial Network (CSP) [28] structure divides the network into segments containing multiple residual blocks, reducing computational load and the number of parameters—crucial for processing large datasets effectively. The YOLOv8 detection head consists of convolutional and pooling layers that process feature maps, which are then converted into precise detection outputs through additional convolutional layers and fully connected layers. This structured approach ensures that each detected pig face is accurately cropped and prepared for the subsequent recognition stage.

YOLO-v8 total loss consists of class loss, objectness loss, and location loss:

(1) $$L_{Total}=\lambda_{1}L_{cls}+\;\lambda_{2}L_{obj}+\lambda_{3}L_{loc},$$

$L_{cls}$: Ensures accurate classification of detected objects into correct categories. $L_{obj}$: Assesses the accuracy in identifying whether a segment contains a pig face. $L_{loc}$: Measures the precision in locating and sizing detected objects.

By leveraging the advanced capabilities of YOLOv8, we effectively reduce the human input required in the preprocessing stages, enhancing the efficiency and scalability of our pig face recognition system. This approach not only conserves resources but also ensures that the system is robust and adaptable to various operational scenarios within smart farming environments.

#### 2.3.2. Pig Face Recognition Module

For the pig face recognition component, we employ a customized Vision Transformer (ViT) architecture designed to function as a robust feature extractor, capable of capturing intricate facial details necessary for accurate identification. Our approach leverages the strengths of pre-trained ViT models while introducing modifications to enhance feature discriminability and robustness against intra-class variations.

**Figure 5:**
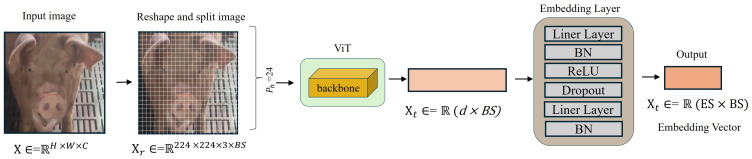
Pig Face Recognition Module in our proposed PFOSR System. This figure illustrates the pig face recognition pipeline in our PFOSR system. The input image is resized to 224 × 224 and processed through a ViT-based backbone for feature extraction. The extracted features are refined through an embedding layer consisting of linear layers, batch normalization (BN), ReLU activation, and dropout, producing the final embedding vector. BS (Batch Size) represents the number of images processed at once, while ES (Embedding Size) refers to the dimensionality of the output feature vector.

The proposed model is designed to extract and refine image features, leveraging the power of Vision Transformer (ViT) architecture and a tailored embedding mechanism to generate highly discriminative feature embeddings as shown in Figure 5. The process begins with the input image, which has a batch shape of 3 × 224 × 224. These images are divided into fixed-size patches of 16 × 16, resulting in a total of 196 patches for each image. Each patch is flattened into a 768 vector of size (16 × 16 × 3). and then linearly projected into a fixed embedding dimension, forming an output tensor of shape (Batch size × 196 × 768). To retain positional information, learnable positional embeddings are added to these patch embeddings, maintaining the shape (Batch size × 196 × 768). This tensor is then passed through the Transformer Encoder, the backbone of ViT, which captures global context and relationships among patches using Multi-Head Self-Attention (MSA) and Feed-Forward Networks (FFN). MSA computes attention weights across patches to derive globally meaningful features, while FFN enhances feature representation through non-linear transformations. Each Transformer Encoder layer includes residual connections and Layer Normalization for stability and convergence. The ViT-Base model contains 12 such layers, processing the input tensor while retaining its shape (Batch size × 196 × 768).

A learnable classification token (CLS token) is prepended to the patch embeddings before entering the Transformer Encoder. After processing, the CLS token, now containing the global representation of the input image, is extracted, resulting in a tensor of shape (Batch size × 768). This serves as the final output of the ViT backbone.

The embedding layer further refines this high-dimensional global feature into a lower-dimensional space suitable for downstream tasks. This layer includes a sequence of transformations: a fully connected layer reduces the feature dimension from 768 to 512, followed by Batch Normalization, ReLU activation, and Dropout for regularization. Another fully connected layer then maps the feature to the target embedding dimension of 64, followed by Batch Normalization, yielding the final feature embeddings of shape (Batch size, 64).

To enhance the discriminative capability of the feature extractor, we adopt a dual-loss training strategy that combines SubCenterArcFace Loss and Center Loss. This approach ensures that the model not only learns to classify known pigs accurately but also maintains robust feature representations that generalize well to unknown individuals, addressing the Open-Set recognition challenge.

(2) $$L_{TotalLoss}=\lambda_{1}L_{\mathrm{SubCenterArcFaceLoss}}+\lambda_{2}L_{\mathrm{CenterLoss}}$$

where $\lambda_{1}$ and $\lambda_{2}$ are hyperparameters that balance the contributions of the classification loss and the metric loss. $\lambda_{1}$ is set to 1, and $\lambda_{2}$ is set to 0.5.

**SubCenterArcFace Loss.** This Loss is an advanced adaptation of the ArcFace Loss, renowned in facial recognition technologies for enhancing the discriminative power of the feature embeddings. The key modification in SubCenterArcFace Loss is the introduction of multiple sub-centers for each class in the feature space. This approach is designed to deal more effectively with intra-class variations by allowing multiple centroids per class, reducing the penalty for intra-class deviations that are still within a reasonable range of the true class center. This flexibility helps to capture a more nuanced representation of each pig’s facial features, accommodating slight variations that are natural among different individuals within the same category.

(3) $$L_{{\mathrm{ArcFace}}_{\mathrm{subcenter}}}=-\log\frac{e^{s\cdot \cos(\theta_{i,y_{i}}+m)}}{e^{s\cdot \cos(\theta_{i,y_{i}}+m)}+\sum_{j=1,j\neq y_{i}}^{N}e^{s\cdot \cos\theta_{i,j}}}$$

(4) $$\theta_{i,j}=\arccos\left(\max_{k}\left(W_{jk}^{⊤}x_{i}\right)\right),\;k\in \left\{1,\ldots ,K\right\}.$$

$\theta_{i,j}$ is the angle between the feature vector associated with the *i*-th example and the weight vector associated with the *j*-th class. arccos means that calculates the angle from the cosine similarity. $\max_{k}$ finds the maximum value among all classes *k*.

**Center Loss.** It complements the metric learning loss by promoting intra-class compactness. It minimizes the distances between feature vectors and their corresponding class centers, ensuring that features of the same pig are tightly clustered in the embedding space. This loss is particularly effective in reducing intra-class variance, thereby enhancing the model’s ability to generalize to new, unseen pigs in Open-Set scenarios. The Center Loss is defined as:

(5) $$L_{\mathrm{Center}}=\sum {i=1}^{N}{\left|x_{i}-c_{y_{i}}\right|}_{2}^{2}$$

where $x_{i}$ is the feature vector of the i-th sample, and $c_{\mathrm{yi}}$.

By combining SubCenterArcFace Loss with Center Loss, our dual-loss structure ensures that the feature extractor not only maximizes inter-class separability but also maintains strong intra-class compactness. This synergy significantly improves the model’s discriminative power, enabling it to capture subtle differences among pig faces while remaining robust to variations within the same class. Consequently, the recognition model achieves high accuracy in both Closed-Set and Open-Set scenarios, effectively addressing the challenges inherent in PFOSR.

### 2.4. Registration and Face Gallery Updating System

This stage involves constructing and maintaining the Pig Face Feature Gallery, enabling the system to adapt to changes in the pig population dynamically. The Pig Face Feature Gallery serves as a repository for feature vectors extracted from images of known pigs, organized as a three-dimensional array with dimensions ($N_{class}$, $N_{Img}$, Embedding vector), where $N_{class}$ represents the number of distinct pig identities (initially 56), $N_{Img}$ denotes the and number of images per pig (30). The embedding vector corresponds to the size of the feature vector extracted by the ViT model (dimension = 768). The gallery’s initial dimensions are (56, 30, 768), representing feature vectors of 56 known pigs.

**Feature Matching.** Our system employs cosine distance to evaluate the similarity between feature vectors during both the new pig registration and inference recognition stages in PFOSR. Cosine similarity measures the cosine of the angle between two vectors in a multi-dimensional space, effectively assessing their similarity regardless of vector magnitude. This metric is ideal for determining whether two feature vectors belong to the same pig.

$$\mathrm{Cos}\mathrm{ine}\;\mathrm{Similarity}=\frac{A\cdot B}{\left|A||B\right|}$$

where *A* and *B* are two pig face feature vectors, A·B denotes the dot product of the vectors, and |A| and |B| are their magnitudes (norms).

**Initial Registration of Known Pigs.** In the initial registration stage, we construct the Pig Face Feature Gallery using the Known Pig Face Feature Dataset. This dataset contains high-quality images of 56 known pigs, with 50 images per pig captured under diverse conditions to ensure robust feature representation. Each image is processed using the pre-trained detection model (YOLOv8) to detect and crop pig faces, and then the ViT feature extractor generates a 768-dimensional feature vector for each pig face. These feature vectors are organized in the feature gallery with dimensions ($N_{class}+1$, $N_{Img}$, 768), where $N_{class}$ = 56, $N_{Img}$ = 30. This gallery serves as the reference for recognizing known pigs during the inference.

**Dynamic Registration of New Pigs.** During the inference, when the system encounters pigs not present in the initial feature gallery, it utilizes the Unknown Pig Face Feature Dataset for dynamic registration. For each pig face detected in the testing dataset, the ViT feature extractor generates a feature vector, which is compared against the existing feature gallery using cosine similarity. If the highest similarity score falls below a predefined threshold (e.g., 0.85), the pig is classified as new. Upon classification as a new pig, the system registers the individual by incorporating additional data from the Unknown Pig Face Feature Dataset. Specifically, 50 images of the new pig are collected under various angles and conditions to capture a comprehensive representation. These images are processed through the detection model and the ViT feature extractor to generate feature vectors, which are then added to the Pig Face Feature Gallery. The gallery is expanded to include the new individual, updating its dimensions to ($N_{class}+1$, $N_{Img}$, 768), where $N_{class}+1$ increments by one for each new pig registered. This dynamic updating mechanism ensures that the system remains current with the changing pig population. By continuously expanding the feature gallery to include new pigs, the system enhances its scalability and efficiency, maintaining high recognition accuracy without the need for re-training. This approach allows the system to adapt to the dynamic nature of farm environments, where new pigs may be introduced regularly.

In summary, the above pipeline of three stages of PFOSR works subsequently during the inference time. In the first stage, the YOLOv8 model detects pig faces in the images, and the detected faces are cropped for the next feature extraction stage. Then the cropped pig face images are input into the ViT model to extract feature vectors. After feature extraction, each feature vector is compared against all vectors in the Pig Face Feature Gallery using cosine similarity. The pig ID corresponding to the highest similarity score is assigned to the detected pig.

## 3. Results

### 3.1. Automatic Pig Face Detection Module

In this section, we detail the implementation of our approach and present both quantitative and qualitative results on the various pig face datasets mentioned in Section 2.2. These evaluations demonstrate the effectiveness of our proposed method in practical pig farming scenarios.

#### 3.1.1. Implementation Details

To develop a robust pig face detection model, we curated a small-scale pig face detection dataset mentioned in Section 2.2 consisting of 1500 high-resolution images of 20 pigs. To ensure the robustness of our model we divide the dataset into training, validation, and testing by setting a 6:2:2 ratio.

We implemented four state-of-the-art object detection models—YOLOv5 [29], YOLOv6 [30], YOLOv7 [31], and YOLOv8—on our annotated pig face dataset. Each model was fine-tuned from pre-trained weights on the MS-COCO dataset to benefit from transfer learning. The training process involved end-to-end optimization using the Stochastic Gradient Descent (SGD) optimizer with a learning rate of 0.01, momentum of 0.937, and weight decay of 0.0005. The original images, with a resolution of 2160 × 3840 pixels, were resized to 224 × 224 pixels to fit the input requirements of the models. Training was conducted for 300 epochs on a system equipped with four NVIDIA Titan GPUs, using a batch size of 64 to optimize computational efficiency. To enhance the models’ generalization capabilities, we applied advanced data augmentation techniques that increase the diversity of the training data. These techniques included Mosaic augmentation, which combines multiple images into one; RandomAffine transformations that apply random affine transformations; Albumentations library for complex augmentations; MixUp, which blends two images; and RandomFlip, which flips images horizontally or vertically. These augmentations help the models learn robust features that are invariant to various transformations.

For evaluating the performance of the detection models, we employed standard object detection metrics, including the ${\mathrm{AP}}_{50–95}$, ${\mathrm{AP}}_{50}$, ${\mathrm{AP}}_{75}$, which averages AP across IoU thresholds from 0.50 to 0.95 in increments of 0.05. and Recall.

#### 3.1.2. Experimental Results

**Training and Evaluation on the dataset of Annotated Small-Scale Pig Face Detection Dataset.**

In our experiments, we assessed the performance of the four YOLO-based object detection models to determine their effectiveness in pig face detection tasks. While YOLOv8 served as our primary detection model due to its advanced architecture and superior performance, the other models provided benchmarks for comparative analysis.

As presented in Table 1, YOLOv8 outperformed its counterparts across all evaluated metrics. It achieved the highest average precisions at IoU thresholds of 0.50 (${\mathrm{AP}}_{50}$ = 0.990), 0.75 (${\mathrm{AP}}_{75}$ = 0.972), and an overall (${\mathrm{AP}}_{50–95}$ = 0.869). Additionally, it attained the highest recall rate of 0.895. These results indicate that YOLOv8 possesses a superior ability to accurately detect pig faces, even under challenging conditions such as varying lighting and occlusions.

**Figure 6:**
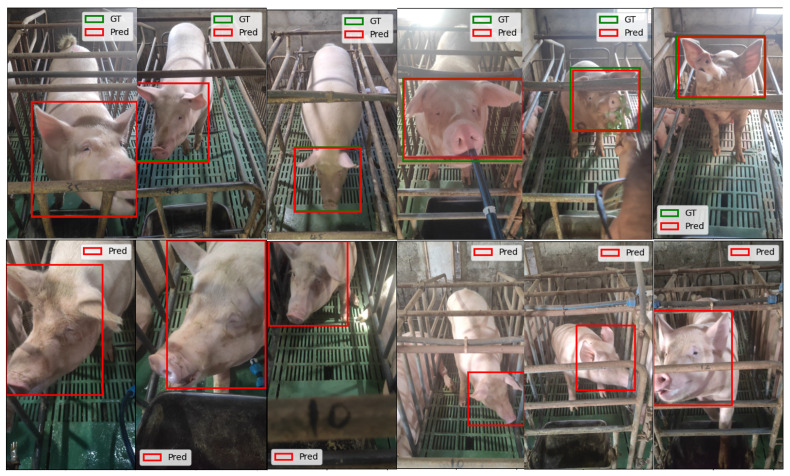
YOLOv8 Performance on Labeled and Unlabeled Datasets. This figure illustrates YOLOv8’s pig face detection across two datasets. In the first row, images from the Small-Scale Pig Face Detection Dataset are shown, with red boxes indicating predictions and blue boxes representing ground truth labels. The second row displays images from the Known Pig Face Recognition Dataset, where red boxes denote YOLOv8’s predictions without ground truth labels. This comparison highlights the model’s effectiveness in both labeled and unlabeled scenarios.

The first row of Figure 6. showcases YOLOv8’s predictions on the test dataset of the Small-Scale Pig Face Detection Dataset. The visualizations highlight the model’s ability to accurately detect pig faces under various conditions, including different poses and lighting environments. This confirms YOLOv8’s effectiveness as the cornerstone of our detection module. Given its outstanding performance, we selected YOLOv8 as the primary detection model for our pig face recognition system. The trained weights from YOLOv8 enable the automated detection of pig faces in unannotated datasets, significantly increasing the amount of training data available for recognition tasks without the need for extensive manual labeling.

#### 3.1.3. Automatic Pig Face Detection Using the Pre-Trained YOLOv8 on Known Pig Face Recognition Dataset

Building on the success of the pre-trained YOLOv8 model, we applied it to automatically detect pig faces in a larger unannotated dataset consisting of images from 56 pigs. This process substantially reduced manual annotation efforts and facilitated the creation of a more comprehensive dataset for the recognition task in the second stage of our system. The second row of Figure 6. presents examples of detected pig faces using the YOLOv8 model on the unannotated dataset. The model effectively identified pig faces under diverse conditions, further demonstrating its utility in preparing data for subsequent recognition tasks.

### 3.2. Pig Face Recognition Module

In this study, we focus on evaluating our pig face recognition model within the framework of Pig Face Open-Set Recognition (PFOSR), which presents unique challenges in distinguishing known pig identities from unknown pigs, previously unseen individuals. The primary objective of our experiments is to systematically assess the model’s performance in both Open-Set and close-set recognition tasks.

#### 3.2.1. Experimental Settings

To achieve these objectives, we constructed four datasets including the Pig Face Gallery, a test set of the Known Pig Face for recognition, the Unknown Pig Face Test Dataset, and 65 known Pig Face Testing Dataset. The gallery dataset serves as a reference database for similarity matching and classification. It contains feature vectors organized by known pig identities, where each identity is represented by multiple embeddings derived from training images. The known test dataset evaluates the model’s recognition performance on individuals included in the gallery, while the unknown test dataset assesses the model’s ability to recognize and reject unfamiliar identities.

All experiments were implemented using the PyTorch framework (version 1.8) and conducted on a server equipped with four NVIDIA GPUs. This computational setup allowed us to train deep learning models with substantial datasets efficiently. We utilized a customized Vision Transformer (ViT) model based on vit-base with 16 patches from the Timm library, which was pre-trained on the ImageNet dataset to leverage transfer learning benefits. Specific modifications were made to the original architecture to better suit the PFOSR task. The models were trained for 200 epochs with a batch size of 128, and gallery size of 30, and input images were resized to 224 × 224 pixels. The embedding size was set to 64 dimensions. An initial learning rate of le-5 was used with the Adam optimizer, employing a step decay schedule to adjust the learning rate during training.

In our proposed dual-loss structure, we utilized Center Loss and SubCenterArcFace Loss as our loss functions, the total loss is a weighted sum of these two losses, with coefficients of 1.0 for the SubCenterArcFace Loss and 0.5 for the Center Loss. The SubCenterArcFace parameters are set to a margin (m) of 28.6, a scale (s) of 64, and 3 sub-centers. This dual-loss approach enhances feature discrimination by simultaneously maximizing inter-class variance and minimizing intra-class variance. Data normalization was performed using mean values of [0.485, 0.456, 0.406] and standard deviations of [0.229, 0.224, 0.225]. To enhance the robustness of the models, we applied data augmentation techniques such as random cropping, horizontal flipping, color jittering, and normalization using the specified mean and standard deviation values. These augmentations help the models generalize better by simulating variations that may occur in real-world scenarios.

#### 3.2.2. Comparative Models

In our main experiment, building upon our improved Vision Transformer (ViT) model, which incorporates several modifications tailored for pig face recognition, we conducted a series of experiments to evaluate the effectiveness of our proposed dual-loss method and transfer learning strategy in Open-Set scenarios. The main objective was to verify how the combination of these components enhances the model’s ability to distinguish between known and unknown pig identities.

In addition, we compared the effectiveness of our proposed dual-loss and transfer learning training strategy with other state-of-the-art models, including ResNet18 [32], ResNet50. These models were evaluated both with and without pre-training to assess the impact of transfer learning. To ensure a fair comparison, the same training parameters and data augmentation strategies were applied across all models.

#### 3.2.3. Evaluation Metrics for the Test Dataset

**Closed-Set Recognition Metrics:** In Closed-Set recognition scenarios, the system is required to classify each input image into one of the known classes. We used the following evaluation metrics:

- Closed-Set Accuracy (CSA) [33]: The proportion of correctly classified images out of the total number of images.
- Precision [34]: The ratio of true positive predictions to the total predicted positives.
- Recall [35]: The ratio of true positive predictions to the total actual positives.
- F1 Score [36]: The harmonic mean of precision and recall.
- Adjusted Mutual Information (AMI) [37]: Measures the agreement between the true labels and the predicted labels, adjusted for chance.
- Normalized Mutual Information (NMI) [38]: Similar to AMI but normalized to a scale between 0 (no mutual information) and 1 (perfect correlation).

**Open-Set Recognition Metrics:** In Open-Set recognition scenarios, the system must identify whether an input image belongs to a known class or is from an unknown class. We used the following metrics:

- Closed-Set Accuracy (CSA) [33]: Calculated only on known classes.
- Area Under the Receiver Operating Characteristic Curve (AUROC) [39]: Measures the model’s ability to distinguish between known and unknown classes.
- Area Under the Precision-Recall Curve (AUPR) [40]: Evaluates the trade-off between precision and recall for different thresholds.
- F1-Open Score [41]: The F1 score adjusted for Open-Set recognition, considering both known and unknown classes.
- False Accept Rate (FAR) [42]: The proportion of unknown class samples that are incorrectly accepted as known classes.
- Correct Classification Rate (CCR) [43]: It measures the percentage of correctly classified instances over all instances, indicating the model’s overall accuracy.
- Open-Set Classification Rate (OSCR) [44]: Combines the correct classification rate of known classes and the false positive rate of unknown classes.

#### 3.2.4. Pig Face Open-Set Recognition Experiments

In the Open-Set scenario, our goal was to determine whether a test image belonged to one of the known pigs in the gallery or was from an unknown pig. To achieve this, we first constructed the Pig Face Feature Gallery by extracting feature embeddings from the images of the Known Pig Face Feature Dataset using the trained models. These embeddings were stored in the Pig Face Gallery, serving as the reference database for identification. For the test images, which included both known pigs (from the testing set of the Known Pig Face Recognition Dataset) and unknown pigs (from the Unknown Pig Face Test Dataset), we extracted their feature embeddings using the same trained models. We then computed the cosine similarity between each test image’s embedding and all embeddings in the Pig Face Gallery. Based on the highest cosine similarity score, we made a threshold-based decision: if the highest similarity score exceeded a threshold of 0.8, the test image was assigned the ID of the corresponding pig with the highest similarity, indicating it was a known pig. If the highest similarity score was below the threshold, the test image was classified as belonging to an unknown pig.

To achieve this goal, we conduct the following experiments. Firstly, We aim to determine the optimal threshold for effectively separating known and unknown classes based on similarity scores. Secondly, we evaluate our model’s performance under this optimal threshold using F1-Open, CCR, and FAR. Then analyze the model’s robustness across varying thresholds using global evaluation metrics like AUROC, AUPR, and OSCR.

**Figure 7:**
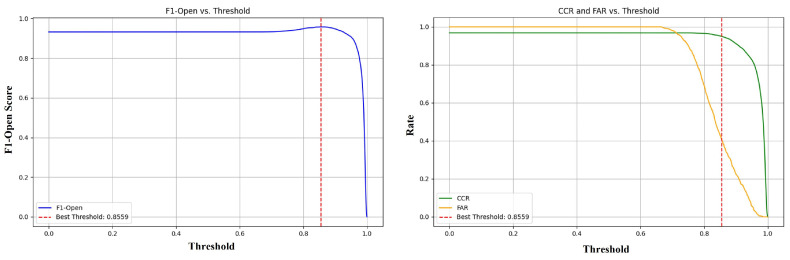
F1-Open, CCR and FAR curves of ViT-DL-IN21K model at different thresholds in PFOSR.

**Best Threshold Experiment for ViT-DL-IN21K.** In our Pig Face Open-Set Recognition (PFOSR) framework, the choice of an optimal threshold is critical for effectively distinguishing between known and unknown classes. The threshold is applied to the cosine similarity between a sample’s embedding and the gallery embeddings, determining whether the sample belongs to a known class or is classified as unknown. To identify the optimal threshold, we utilized F1-Open, CCR, and FAR as evaluation metrics. F1-Open reflects the model’s ability to balance precision and recall in classifying known classes while rejecting unknown ones. CCR measures the proportion of correctly classified known samples, while FAR indicates the proportion of unknown samples mistakenly classified as known. These metrics collectively ensure that the selected threshold achieves a balance between recognizing known classes and rejecting unknown ones. Our results, visualized in Figure 7, demonstrate how F1-Open, CCR, and FAR vary with the threshold. The F1-Open curve initially increases with higher thresholds as the model becomes better at rejecting unknown classes but declines beyond a certain point due to the rejection of known samples. At the threshold of 0.8559, F1-Open reaches its maximum value, identifying this as the optimal threshold. Similarly, the CCR curve decreases with increasing thresholds as known samples are gradually rejected, while the FAR curve shows a steep decline, indicating improved rejection of unknown samples. At 0.8559, CCR remains high at 95.03%, and FAR is minimized at 40.33%, achieving the best trade-off. Based on these findings, we used 0.8559 as the threshold for recognizing and registering unknown classes, with the details of the registration mechanism provided in the 2.5 section discussing Open-Set classification and registration.

**Impact of Dual loss functions and transfer learning on PFOSR Performance.** To assess the impact of dual loss functions and transfer learning on Pig Face Open-Set Recognition (PFOSR) performance, we conducted a comprehensive evaluation of our ViT-DL-IN21K model. This model is based on our proposed improved ViT architecture, pre-trained on the extensive ImageNet-21K dataset. We fine-tuned this model on our specific pig face dataset, implementing a dual-loss training strategy (DL) that combines Sub-Center ArcFace Loss (SAL) and Center Loss (CL). Sub-Center ArcFace Loss enhances robustness against noisy data by assigning multiple sub-centers to each class, allowing the model to capture complex intra-class variations. Center Loss, on the other hand, minimizes the distance between features and their corresponding class centers, promoting intra-class compactness. By integrating these loss functions, our dual-loss approach aims to improve the model’s ability to distinguish between known and unknown pig faces. Additionally, we compared the performance of models trained from scratch with those fine-tuned from pre-trained weights to evaluate the benefits of transfer learning. This analysis provides insights into how the choice of loss functions and transfer learning strategies influence PFOSR performance. Table 2 summarizes the performance of various Vision Transformer (ViT) models on the Unknown Pig Face Test Dataset, using a gallery size of 50. The results demonstrate that models trained with the dual-loss method consistently outperform those trained with SAL or CL alone, with ViT-DL achieving better metrics than ViT-SAL and ViT-CL, showing the dual-loss method’s ability to balance inter-class separability and intra-class compactness, thus generating more discriminative features. Pre-training on ImageNet-21K further improves performance across all models, as shown by the superior results of ViT-SAL-IN21k and ViT-CL-IN21k compared to their counterparts trained from scratch, indicating that pre-trained models provide a stronger feature extraction foundation. Combining the dual-loss method with Pre-training, our ViT-DL-IN21K model achieves the best overall performance, with a CSA of 96.60%, AUROC of 95.31%, OSCR of 95.87%, AUPR of 99.30%, and an F1-Open score of 93.77%. These results highlight the synergistic effects of the dual-loss method and pre-training, with the dual loss optimizing both inter-class separability and intra-class compactness, while pre-training delivers robust feature initialization. Together, these strategies make ViT-DL-IN21K the most effective model for Pig Face Open-Set Recognition, excelling in both Open-Set and Closed-Set scenarios.

**Impact of Gallery Size on PFOSR Performance**. We examined the effect of varying the gallery size on the recognition performance of the ViT-DL-IN21K model. The gallery size refers to the number of images per pig used to construct the feature gallery, ranging from 10 to 50 images in increments of 10. By altering the gallery size, we aimed to understand how the amount of reference data influences the model’s ability to correctly identify pigs and detect unknown individuals in an Open-Set scenario. We examined the effect of varying the gallery size on the recognition performance of the ViT-DL-IN21K model in Pig Face Open-Set Recognition (PFOSR). The gallery size refers to the number of images per pig used to construct the feature gallery, ranging from 10 to 50 images in increments of 10. Adjusting the gallery size alters the number of stored features, which can influence both the recognition accuracy and the computational efficiency during testing. A smaller gallery size reduces storage requirements and speeds up testing but may compromise recognition performance due to fewer reference features. Conversely, a larger gallery size provides more reference features, potentially improving recognition accuracy but increasing storage needs and computational time. Therefore, identifying an optimal gallery size is crucial for balancing performance and efficiency. Table 3 presents the performance metrics of the ViT-DL-IN21K model with different gallery sizes. From the results in Table 3, we observe that increasing the gallery size from 10 to 30 images per pig leads to improvements across all evaluation metrics. The Closed-Set Accuracy (CSA) increases from 95.30% to 96.88%, indicating enhanced recognition of known pigs. The AUROC and AUPR values also improve, reflecting better discrimination between known and unknown pigs. The OSCR and F1-Open scores peak at a gallery size of 30, suggesting an optimal balance between correct classification and rejection of unknown classes. Beyond a gallery size of 30 images per pig, the performance gains plateau. The metrics for gallery sizes of 40 and 50 are similar to those at 30, with slight fluctuations within the margin of error. This indicates that adding more images per pig beyond 30 does not yield significant improvements in recognition performance. Moreover, larger gallery sizes increase storage requirements and computational time during testing, which can be detrimental in practical applications where efficiency is essential. Therefore, a gallery size of 30 images per pig appears to provide the optimal trade-off between recognition accuracy and computational efficiency. It offers sufficient reference data to maximize the model’s performance without incurring unnecessary storage and processing overhead.

**Comparison of state-of-art-of-art Models Using the dual-loss Method**. To evaluate the generality and effectiveness of our dual-loss method across different architectures, we applied it to ResNet18, ResNet50, and ViT−Thin models. Table 4 demonstrates the effectiveness of our dual-loss method across different architectures, including ResNet18, ResNet50, and ViT-Thin. The dual-loss method consistently improved Open-Set recognition metrics, such as CSA, AUROC, OSCR, AUPR, and F1-Open, compared to models trained with SubCenterArcFace Loss (SAL) alone. For ResNet models, ResNet50−DL achieved the most notable gains, with an OSCR of 94.68 and an AUPR of 96.76, highlighting the dual-loss method’s ability to enhance feature discrimination. While ResNet-based models benefited significantly from the dual-loss strategy, the ViT-DL-IN21K model demonstrated the strongest overall performance, leveraging its transformer−based architecture to better capture complex relationships and extract discriminative embeddings. These results validate the generality of the dual-loss method while emphasizing the superior capability of transformers for Open-Set recognition in pig face recognition tasks.

#### 3.2.5. Performance on the Pig Face Closed-Set Recognition (PFCSR)

To comprehensively evaluate the Closed-Set recognition capabilities of our models, we compared ResNet18-DL-IN21K, ResNet50-DL-IN21K, and ViT-DL-IN21K across two distinct test datasets. Dataset1 refers to the test set from the Known Pig Face Recognition Dataset, which includes images of 56 pigs. Dataset2 is a combination of Dataset1 and the Unknown Pig Face Test Dataset, resulting in a comprehensive set that encompasses images of both the 56 known pigs and 9 additional unknown pigs. This dual-test approach allowed us to assess the models’ performance under ideal and more challenging conditions, where unknown classes exist.

Table 5 presents the Closed-Set recognition performance metrics of different models on two datasets using the Pig Face Gallery. All models evaluated—Res18-DL-IN21k, Res50-DL-IN21k, and ViT-DL-IN21K—incorporate our proposed dual-loss structure and leverage transfer learning by being pre-trained on ImageNet21k. Despite using the same training methodology, the ViT model demonstrates superior performance due to its architectural advantages. On Dataset1, which contains 56 known pig classes, the ViT-DL-IN21K model achieves an AMI of 97.06%, NMI of 97.26%, CSA of 96.60%, P-R of 95.10%, MAP@R of 94.64%, and F1-Score of 97.28%. In comparison, the Res50-DL-IN21k model attains an AMI of 93.73%, NMI of 94.17%, CSA of 94.27%, P-R of 92.04%, MAP@R of 91.42%, and F1-Score of 94.94%. The Res18-DL-IN21k model shows slightly lower performance than Res50-DL-IN21k. These results indicate that while all models benefit from the dual-loss structure and pre-training, the ViT architecture provides a significant performance boost. On Dataset2, which includes both known and newly registered unknown classes (totaling 65 pig classes), ViT-DL-IN21K maintains high performance with an AMI of 94.72%, NMI of 95.05%, CSA of 93.59%, P-R of 86.93%, MAP@R of 85.32%, and F1-Score of 92.93%. The Res50-DL-IN21k model achieves an AMI of 89.14%, NMI of 89.82%, CSA of 87.29%, P-R of 87.29%, MAP@R of 79.99%, and F1-Score of 85.76%. Again, the ViT model outperforms the ResNet models, showing a smaller decrease in performance when handling newly registered classes without retraining.

These results demonstrate that although all models employ our dual-loss structure and benefit from transfer learning, the Vision Transformer (ViT) architecture significantly enhances recognition performance. The superior results of ViT-DL-IN21K suggest that the ViT model is more effective in capturing discriminative features for pig face recognition, particularly in Open-Set scenarios. This validates our approach in dynamic farm environments and confirms the suitability of the ViT architecture for handling the complexities of pig face recognition tasks.

**Figure 8:**
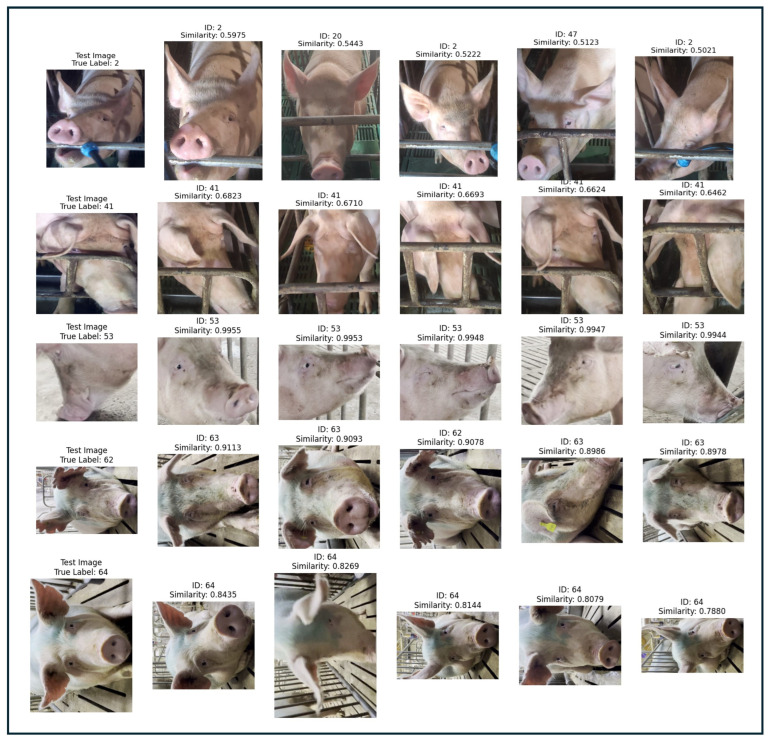
Visualization of ViT-DL-IN21K Model Performance on the 65 known Pig Face Testing Dataset. Each panel displays a test image with its true ID, followed by the top five most similar gallery images with their cosine similarity scores and true IDs. High similarity scores indicate correct matches for known classes, including newly registered ones.

To further demonstrate the efficacy of our ViT-DL-IN21K model in Closed-Set recognition, we present a visual analysis of its performance on the expanded test set comprising 65 pig faces (56 known and 9 previously unknown classes that have been successfully registered). Figure 8. illustrates several test images alongside their true identities and the top five most similar matches retrieved from the Pig Face Gallery. Each retrieved image is accompanied by its cosine similarity score and corresponding true ID. In the first column of Figure 8, the test image is accurately matched with gallery images that share the same true ID, evidenced by high similarity scores ranging from 0.85 to 0.96. This consistent matching across different test samples highlights the model’s robust discriminative capabilities, ensuring that each known class is correctly identified and associated with the gallery database. The high similarity scores confirm that the feature representations learned by the ViT- DL-IN21k model are both distinctive and reliable, effectively capturing the nuances of each pig face. Moreover, the successful matching of test images from the previously unknown classes, now registered as known, underscores the effectiveness of our registration mechanism. By incorporating these new classes into the gallery, the model seamlessly integrates them into the Closed-Set recognition framework, maintaining high accuracy and minimizing misclassifications. This visualization not only validates the model’s ability to handle dynamic class additions but also reinforces its suitability for real-world applications in intelligent pig farming, where the ability to recognize and adapt to new individuals is crucial.

**Figure 9:**
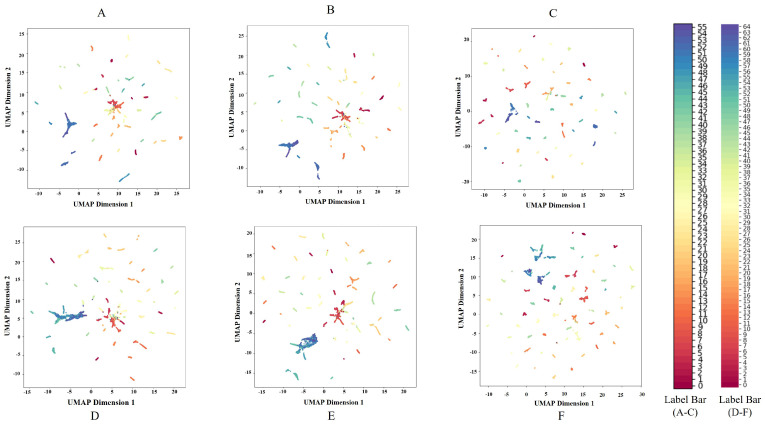
UMAP Visualization of Test Features for Model Performance on Dataset1 and Dataset2 in PFOSR System. This figure presents the UMAP projections of test feature embeddings for three models (ResNet18-DL-IN21K, ResNet50-DL-IN21K, and ViT-DL-IN21K) on two datasets. (**A**–**C**) correspond to Dataset1 (Known Pig Face Recognition Dataset test set, 56 known classes), while (**D**–**F**) correspond to Dataset2 (65-Known Pig Face Testing Dataset, including 56 known classes and 9 newly registered unknown classes). Each color represents a different pig identity. A more compact and well-separated clustering indicates better feature representation. ViT-DL-IN21K (**C**,**F**) shows improved feature clustering, demonstrating its superior ability to distinguish different pig identities, especially in Dataset2, where new classes have been introduced.

To further demonstrate the robust Closed-Set recognition capabilities of our ViT-DL-IN21K model, we employed Uniform Manifold Approximation and Projection (UMAP) to visualize the high-dimensional feature embeddings of the expanded test set, which includes 65 pig faces (56 known and 9 newly registered classes). Figure 9 presents the UMAP projection, where each point represents an image embedding, color-coded by its true class ID. For Dataset1, with 56 known classes, the ViT-based model exhibits superior performance compared to the ResNet models. Its feature clusters are well-separated, demonstrating clear class boundaries and robust feature extraction. In contrast, while ResNet50 shows reasonably tight clusters, there is a slightly higher degree of overlap in some class boundaries. ResNet18, being a smaller model, displays the least compact clusters among the three, with noticeable dispersion in certain class features, indicating potential challenges in class separability. In the extended Dataset2 containing 65 classes, including unknown classes, the ViT model again outperforms the ResNet models. The unknown classes are better distributed and show less overlap with the known classes, highlighting the ViT model’s ability to generalize to unseen data. ResNet50, while effective in handling the known classes, struggles with the unknown classes, showing more significant overlap in the feature space. ResNet18 faces similar issues but to a greater extent, with both known and unknown classes displaying less distinct clusters. Overall, the ViT-based model, as the proposed method, demonstrates the best performance across both datasets, with well-separated features and effective handling of unknown classes. This suggests that the ViT model is better suited for tasks requiring strong generalization and robust feature representation in complex scenarios.

**Figure 10:**
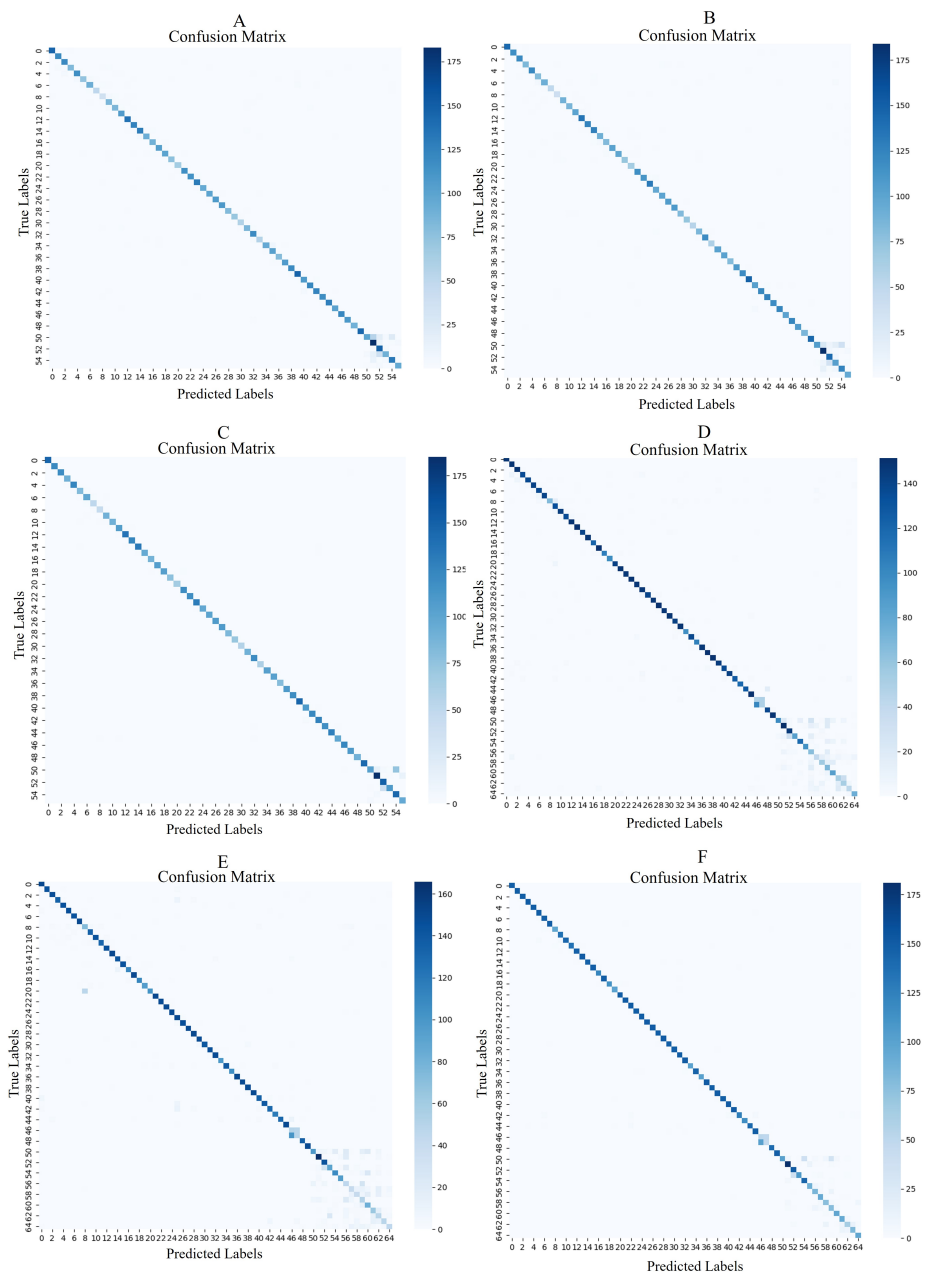
Confusion Matrices for Models Performance on testing Dataset1 and testing Dataset2 in PFCSR System. This figure presents the confusion matrices for three models (ResNet18-DL-IN21K, ResNet50-DL-IN21K, and ViT-DL-IN21K) evaluated on two test datasets. (**A**–**C**) correspond to Dataset1 (Known Pig Face Recognition Dataset test set, 56 known classes), while (**D**–**F**) correspond to Dataset2 (65-Known Pig Face Testing Dataset, including 56 known classes and 9 newly registered unknown classes). The diagonal elements represent correct classifications, while off-diagonal elements indicate misclassification. Comparing these matrices, ViT-DL-IN21K (**C**,**F**) achieves the lowest misclassification rates, as seen by the darker diagonal and lighter off-diagonal cells. This demonstrates its superior performance in both standard Closed-Set recognition (Dataset1) and recognizing newly registered pigs in Dataset2, proving its robustness and adaptability in pig face recognition.

In Figure 10, the confusion matrices demonstrate the superior performance of the ViT-based model across both datasets. For the 56-class dataset, it shows minimal misclassifications, outperforming ResNet50 and ResNet18. In the extended 65-class dataset, the ViT model effectively distinguishes unknown classes, with fewer errors than the ResNet models. ResNet50 performs well but shows slight confusion, while ResNet18 struggles more with class separability and unknown classes. Overall, the ViT model excels in accuracy and generalization, proving most effective for pig face Closed-Set recognition tasks.

Table 6 illustrates the impact of different gallery sizes on the performance metrics of the ViT-DL-IN21K model with the testing dataset of the Known Pig Face Recognition Dataset (56 class) in Closed-Set recognition tasks. As the gallery size increases from 10 to 50, the model maintains consistently high performance across all evaluated metrics, with only minimal variations. When the gallery size is 10, the model achieves a CSA of 96.44%, Precision of 97.16%, Recall of 97.20%, and F1-Score of 96.99%. With the gallery size increased to 30, these metrics slightly improve, reaching a CSA of 96.76%, Precision of 97.53%, Recall of 97.55%, and F1-Score of 97.36%. Notably, the model attains its best performance with a gallery size of 30, indicating an optimal balance between recognition accuracy and computational efficiency. Using a gallery size of 30 not only enhances performance but also reduces computational cost during testing compared to larger gallery sizes. These results demonstrate that the ViT-DL-IN21K model exhibits excellent stability and generalization ability across different gallery sizes, making it reliable for practical applications where the number of registered pig faces may vary.

## 4. Discussion

This study establishes a high-performing three-stage pipeline that integrates advanced pig face detection and recognition techniques with a dynamic registration mechanism, effectively addressing key challenges in dynamic livestock farming environments. The pig face detection stage is implemented using YOLOv8, which achieves state-of-the-art performance on the Small-Scale Pig Face Detection Dataset. The model achieved an ${\mathrm{AP}}_{50}$ of 0.990, an ${\mathrm{AP}}_{75}$ of 0.972, and an overall ${\mathrm{AP}}_{50–95}$ of 0.869, surpassing other YOLO variants such as YOLOv5, YOLOv6, and YOLOv7. These metrics demonstrate the model’s ability to accurately localize pig faces, even under challenging conditions, including varying lighting, occlusions, and complex backgrounds. Additionally, the recall rate of 0.895 further underscores the system’s effectiveness in detecting pig faces, significantly minimizing missed detections.

The second stage of the system, focusing on pig face recognition, utilizes a modified Vision Transformer (ViT) architecture optimized with a dual-loss strategy combining SubCenterArcFace Loss and Center Loss. This model achieved a Closed-Set recognition accuracy (CSA) of 96.60% on the known Pig Face Test Dataset. A key feature of the proposed system is the dynamic registration mechanism, allowing the seamless integration of new pigs into the feature gallery without the need to retain new pigs. The dynamic gallery, which originally contained 56 pigs, was assessed by gradually introducing 9 additional unknown pigs. The system upheld its high performance, achieving an updated AUROC of 94.72% and an F1-Open score of 92.93%, demonstrating its ability to adapt to changes in population size. The gallery size was systematically analyzed to optimize performance. A gallery size of 30 images per pig achieved the best trade-off between computational efficiency and recognition accuracy, with a CSA of 96.88%, AUROC of 95.31%, and OSCR of 95.87% in PFOSR and CSA OF 96.76%, precision of 97.53%, NMI of 97.32%, AMI of precision@1 of 96.76% in PFCSR task. But beyond 30 images, performance gains plateaued. The practical implications of these findings are significant. The system’s ability to achieve high precision in Closed-Set recognition and strong performance in Open-Set scenarios makes it a valuable tool for modern livestock management. By automating the identification and monitoring of pigs, the system reduces labor costs, improves record-keeping accuracy, and enhances animal welfare. Additionally, its dynamic registration mechanism ensures that the system remains operational in real-time, even as the farm population changes, addressing a critical gap in the existing animal recognition system.

Despite these achievements, the study reveals areas for improvement. While the proposed system performs well on specific datasets, its scalability to larger herds remains untested. Larger datasets could potentially introduce computational bottlenecks in real-time operations, necessitating further optimization of the feature-matching process. Expanding the dataset to include diverse breeds and more complex farm environments would enhance the system’s generalizability.

## 5. Conclusions

This study presents a robust three-stage system for pig face detection, recognition, and dynamic registration, designed to address the challenges of managing livestock in dynamic farming environments. The pig face detection module utilizes the YOLOv8 model and a modified Vision Transformer with a dual-loss strategy for accurate pig face recognition tasks. The system achieves state-of-the-art performance in both Closed-Set and Open-Set recognition tasks. The inclusion of a dynamic registration mechanism ensures adaptability to population changes without requiring or retraining the pig, making the system highly scalable and efficient. With high metrics such as an AUROC of 95.31% and an F1-Open score of 93.77%, the system demonstrates its effective performance for the pig face open set recognition (PFOS) task. The limitations in this work can be addressed in future works by incorporating dataset diversity and scalability to larger herds. This research work marks a significant advancement in the field of precision livestock management. It introduces a non-invasive and highly accurate method for monitoring and managing individual animals, which is both scalable and practical for widespread use. This innovative approach enables farmers and livestock managers to track various health and performance metrics of each animal without causing stress or disruption to their natural behaviors. By utilizing advanced technologies, this solution improves animal welfare and enhances overall productivity, paving the way for more sustainable and efficient livestock farming practices.

## Figures and Tables

**Table 1:** Performance metrics of different detection models on the Testing dataset of Small-Scale Pig Face Detection Dataset.

Model	${\mathrm{AP}}_{50}$	${\mathrm{AP}}_{75}$	${\mathrm{AP}}_{50–95}$	Recall
YOLOV-5	0.975	0.955	0.821	0.779
YOLOV-6	0.986	0.969	0.861	0.865
YOLOV-7	0.980	0.964	0.840	0.876
YOLOV-8	**0.990**	**0.973**	**0.869**	**0.895**

Note: The values that recorded the highest performance for each metric are highlighted in bold.

**Table 2:** Performance comparison of ViT-based models on the Testing Set of Known Pig Face Recognition Dataset using Pig Face Gallery.

Model	Training Strategies	CSA	AUROC	OSCR	AUPR	F1-Open
ViT-SAL	-	94.02 ± 0.5	92.71 ± 0.2	92.71 ± 1.8	97.33 ± 0.5	89.81 ± 0.4
ViT-SAL-IN21k	✔	93.38 ± 1.1	92.95 ± 1.1	93.75 ± 1.1	98.98 ± 0.3	92.58 ± 0.2
ViT-CL	-	91.69 ± 0.2	90.14 ± 0.8	91.20 ± 0.2	95.13 ± 0.4	85.11 ± 1.6
ViT-CL-IN21k	✔	92.25 ± 0.7	91.70 ± 0.4	91.81 ± 0.6	95.77 ± 0.7	86.63 ± 1.3
ViT-DL	-	95.25 ± 0.7	94.26 ± 1.1	94.21 ± 0.1	98.77 ± 0.3	92.63 ± 0.3
ViT-DL-IN21K (our)	✔	**96.60** ± 0.4	**95.31** ± 0.2	**95.87** ± 0.5	**99.30** ± 0.1	**93.77** ± 0.2

SAL: SubCenterArcFace Loss; CL: Center Loss; DL: dual-loss; IN21K: ImageNet-21K. “✔”: symbol in the “Training Strategies” column denotes models that leverage transfer learning from ImageNet-21K, while “-” indicates models trained from scratch. Note: The values that recorded the highest performance for each metric are highlighted in bold.

**Table 3:** Impact of gallery size on PFOSR performance of the ViT-DL-IN21K model.

Size of Gallery	CSA	AUROC	OSCR	AUPR	F1-Open
10	95.30 ± 1.1	94.18 ± 0.4	92.01 ± 0.4	98.77 ± 0.6	92.41 ± 0.1
20	95.71 ± 0.2	95.14 ± 0.4	93.86 ± 0.2	99.11 ± 0.5	92.51 ± 0.1
30	**96.88 ± 0.4**	**95.31 ± 0.2**	95.87 ± 0.5	**99.30 ± 0.1**	**93.77 ± 0.2**
40	96.84 ± 0.1	95.21 ± 0.6	95.76 ± 0.1	99.29 ± 0.2	93.71 ± 0.4
50	96.00 ± 0.5	95.18 ± 1.7	**95.91 ± 0.3**	99.23 ± 0.2	93.16 ± 1.2

Note: The values that recorded the highest performance for each metric are highlighted in bold.

**Table 4:** Performance metrics of different models on the testing set of Known Pig Face Recognition Dataset using Pig Face Gallery.

Model	CSA	AUROC	OSCR	AUPR	F1-Open
Res18−SAL	90.91	89.24	90.16	94.37	83.74
Res18-DL-IN21k (our)	93.98	90.02	91.11	93.17	84.12
Res50−SAL	93.47	91.55	92.36	94.58	84.24
Res50-DL-IN21k (our)	**94.27**	**92.33**	**94.68**	**96.76**	**86.10**

Note: The values that recorded the highest performance for each metric are highlighted in bold.

**Table 5:** Closed-Set recognition performance metrics of different models on the different datasets using Pig Face Gallery.

Dataset	Model	AMI	NMI	CSA	P-R	MAP@R	F1-Score	Precision@1
Dataset1	Res18-DL-IN21k	93.63 ± 1.1	94.04 ± 0.3	93.98 ± 0.4	92.80 ± 0.6	92.09 ± 0.1	94.64 ± 0.4	93.98 ± 0.3
	Res50-DL-IN21k	93.73 ± 0.2	94.17 ± 0.7	94.27 ± 0.1	92.04 ± 0.6	91.42 ± 0.1	94.94 ± 0.5	94.27 ± 0.2
	ViT-DL-IN21K	**97.06** ± 0.4	**97.26** ± 0.2	**96.60** ± 0.6	**95.10** ± 0.1	**94.64** ± 0.1	**97.28** ± 0.1	**96.60** ± 0.1
Dataset2	Res18-DL-IN21k	89.25 ± 1.2	89.92 ± 0.4	88.09 ± 0.5	82.16 ± 0.7	80.28 ± 0.2	86.80 ± 0.1	88.09 ± 0.4
	Res50-DL-IN21k	89.14 ± 0.3	89.82 ± 0.6	87.29 ± 0.2	87.29 ± 0.8	79.99 ± 0.8	85.76 ± 0.2	87.26 ± 0.3
	ViT-DL-IN21K	**94.72** ± 0.5	**95.05** ± 0.3	**93.587** ± 0.5	**86.93** ± 0.4	**85.32** ± 0.1	**92.93** ± 0.1	**93.58** ± 0.4

Dataset1: Test dataset of Known Pig Face Recognition Dataset. Dataset2: 65 Known Testing Dataset. Note: The values that recorded the highest performance for each metric are highlighted in bold.

**Table 6:** Impact of gallery size on PFCSR performance of the ViT-DL-IN21K model with the testing dataset of Known Pig Face Recognition Dataset (56 class).

Gallery Size	CSA	Precision	Recall	F1-Score	NMI	AMI	Precision@1	R-P	MAP@R
10	96.44	97.16	97.20	96.99	96.98	96.76	96.44	95.43	95.12
20	96.64	97.48	97.40	97.26	97.29	97.08	96.64	95.24	94.84
30	**96.76**	**97.53**	**97.55**	**97.36**	**97.32**	**97.11**	**96.76**	**95.34**	**94.93**
40	96.54	97.48	97.39	97.22	97.19	96.97	96.54	95.02	94.56
50	96.60	**97.53**	97.47	97.28	97.26	97.06	96.60	95.10	94.64

Note: The values that recorded the highest performance for each metric are highlighted in bold.

## Data Availability

The data presented in this study are available in the article.

## Supplementary Materials

The following supporting information can be downloaded at: https://www.mdpi.com/article/10.3390/ani15050691/s1. Figure S1. The visualization of pig face annotation using LabelMe Tool.

## References

[1] Mahfuz S., Mun H.S., Dilawar M.A., Yang C.J. (2022). Applications of smart technology as a sustainable strategy in modern swine farming. Sustainability.

[2] Zhang C., Lu Y. (2021). Study on artificial intelligence: The state of the art and future prospects. J. Ind. Inf. Integr..

[3] Marsot M., Mei J., Shan X., Ye L., Feng P., Yan X., Li C., Zhao Y. (2020). An adaptive pig face recognition approach using Convolutional Neural Networks. Comput. Electron. Agric..

[4] Adrion F., Kapun A., Eckert F., Holland E.M., Staiger M., Götz S., Gallmann E. (2018). Monitoring trough visits of growing-finishing pigs with UHF-RFID. Comput. Electron. Agric..

[5] Maselyne J., Saeys W., Briene P., Mertens K., Vangeyte J., De Ketelaere B., Hessel E.F., Sonck B., Van Nuffel A. (2021). Methods to construct feeding visits from RFID registrations of growing-finishing pigs at the feed trough. Comput. Electron. Agric..

[6] Li L., Mu X., Li S., Peng H. (2020). A review of face recognition technology. IEEE Access.

[7] Dong S., Wang P., Abbas K. (2021). A survey on deep learning and its applications. Comput. Sci. Rev..

[8] Billah M., Wang X., Yu J., Jiang Y. (2022). Real-time goat face recognition using convolutional neural network. Comput. Electron. Agric..

[9] Weng Z., Meng F., Liu S., Zhang Y., Zheng Z., Gong C. (2022). Cattle face recognition based on a Two-Branch convolutional neural network. Comput. Electron. Agric..

[10] Wan Z., Tian F., Zhang C. (2023). Sheep face recognition model based on deep learning and bilinear feature fusion. Animals.

[11] Meng Y., Yoon S., Han S., Fuentes A., Park J., Jeong Y., Park D.S. (2023). Improving Known–Unknown Cattle’s Face Recognition for Smart Livestock Farm Management. Animals.

[12] Zhang X., Xuan C., Xue J., Chen B., Ma Y. (2023). LSR-YOLO: A high-precision, lightweight model for sheep face recognition on the mobile end. Animals.

[13] Wang H., Qin J., Hou Q., Gong S. (2020). Cattle face recognition method based on parameter transfer and deep learning. J. Phys. Conf. Ser..

[14] Wang R., Shi Z., Li Q., Gao R., Zhao C., Feng L. (2021). Pig face recognition model based on a cascaded network. Appl. Eng. Agric..

[15] Yan L., Miao Z., Zhang W. (2022). Pig face detection method based on improved CenterNet algorithm. Proceedings of the 2022 3rd International Conference on Electronic Communication and Artificial Intelligence (IWECAI).

[16] Hansen M.F., Smith M.L., Smith L.N., Salter M.G., Baxter E.M., Farish M., Grieve B. (2018). Towards on-farm pig face recognition using convolutional neural networks. Comput. Ind..

[17] Wang Z., Liu T. (2022). Two-stage method based on triplet margin loss for pig face recognition. Comput. Electron. Agric..

[18] Ma R., Ali H., Chung S., Kim S.C., Kim H. (2023). A lightweight pig face recognition method based on automatic detection and knowledge distillation. Appl. Sci..

[19] Wang R., Gao R., Li Q., Dong J. (2023). Pig face recognition based on metric learning by combining a residual network and attention mechanism. Agriculture.

[20] Musgrave K., Belongie S., Lim S.N. (2020). Pytorch metric learning. arXiv.

[21] Deng J., Guo J., Xue N., Zafeiriou S. Arcface: Additive angular margin loss for deep face recognition. Proceedings of the IEEE/CVF Conference on Computer Vision and Pattern Recognition.

[22] Wen Y., Zhang K., Li Z., Qiao Y. (2016). A discriminative feature learning approach for deep face recognition. Proceedings of the European Conference on Computer Vision.

[23] Alexey D. (2020). An image is worth 16x.16 words: Transformers for image recognition at scale. arXiv.

[24] Deng J., Guo J., Liu T., Gong M., Zafeiriou S. (2020). Sub-center arcface: Boosting face recognition by large-scale noisy web faces. Computer Vision—ECCV 2020, Proceedings of the 16th European Conference, Glasgow, UK, 23–28 August 2020.

[25] Xia P., Zhang L., Li F. (2015). Learning similarity with cosine similarity ensemble. Inf. Sci..

[26] Jocher G., Chaurasia A., Qiu J. (2023). YOLO by Ultralytics. https://github.com/ultralytics/ultralytics.

[27] Guo X., Jiang F., Chen Q., Wang Y., Sha K., Chen J. (2024). Deep Learning-Enhanced Environment Perception for Autonomous Driving: MDNet with CSP-DarkNet53. Pattern Recognit..

[28] Wang C.Y., Bochkovskiy A., Liao H.Y.M. Scaled-yolov4: Scaling cross stage partial network. Proceedings of the IEEE/CVF Conference on Computer Vision and Pattern Recognition.

[29] Jocher G., Chaurasia A., Stoken A., Borovec J., Kwon Y., Michael K., Fang J., Wong C., Zeng Y., Mammana L. (2022). ultralytics/yolov5: V6. 2-yolov5 classification models, apple m1, reproducibility, clearml and deci. ai integrations. Zenodo.

[30] Li C., Li L., Jiang H., Weng K., Geng Y., Li L., Ke Z., Li Q., Cheng M., Wei X. (2022). YOLOv6: A single-stage object detection framework for industrial applications. arXiv.

[31] Wang C.Y., Bochkovskiy A., Liao H.Y.M. YOLOv7: Trainable bag-of-freebies sets new state-of-the-art for real-time object detectors. Proceedings of the IEEE/CVF Conference on Computer Vision and Pattern Recognition.

[32] He K., Zhang X., Ren S., Sun J. Deep residual learning for image recognition. Proceedings of the IEEE Conference on Computer Vision and Pattern Recognition.

[33] Vaze S., Han K., Vedaldi A., Zisserman A. (2021). Open-Set recognition: A good Closed-Set classifier is all you need?. arXiv.

[34] Streiner D.L., Norman G.R. (2006). “Precision” and “accuracy”: Two terms that are neither. J. Clin. Epidemiol..

[35] Fränti P., Mariescu-Istodor R. (2023). Soft precision and recall. Pattern Recognit. Lett..

[36] Yacouby R., Axman D. Probabilistic extension of precision, recall, and f1 score for more thorough evaluation of classification models. Proceedings of the First Workshop on Evaluation and Comparison of NLP Systems.

[37] Zhu Z., Gao Y. (2022). Finding cross-border collaborative centres in biopharma patent networks: A clustering comparison approach based on adjusted mutual information. Complex Networks & Their Applications X: Volume 1, Proceedings of the Tenth International Conference on Complex Networks and Their Applications COMPLEX NETWORKS, Palermo, Italy, 8–10 November.

[38] Kvålseth T.O. (2017). On normalized mutual information: Measure derivations and properties. Entropy.

[39] Neal L., Olson M., Fern X., Wong W., Li F. Open set learning with counterfactual images. Proceedings of the European Conference on Computer Vision (ECCV).

[40] Huang H., Wang Y., Hu Q., Cheng M.M. (2022). Class-specific semantic reconstruction for open set recognition. IEEE Trans. Pattern Anal. Mach. Intell..

[41] Kozerawski J., Turk M. (2021). One-class meta-learning: Towards generalizable few-shot Open-Set classification. arXiv.

[42] Yum D.H., Kim J.S., Hong S.J., Lee P.J. (2021). Distance bounding protocol with adjustable false acceptance rate. IEEE Commun. Lett..

[43] Adachi K. (2004). Correct classification rates in multiple correspondence analysis. J. Jpn. Soc. Comput. Stat..

[44] Kim S., Kim H.I., Ro Y.M. Improving open set recognition via visual prompts distilled from common-sense knowledge. Proceedings of the AAAI Conference on Artificial Intelligence.

