# Supplementary figures and images for: Pig Face Open Set Recognition and Registration Using a Decoupled Detection System and Dual-Loss Vision Transformer
Source: Animals (Basel). 2025 Feb 27;15(5):691. doi: 10.3390/ani15050691 (PMC11898941; doi:10.3390/ani15050691)

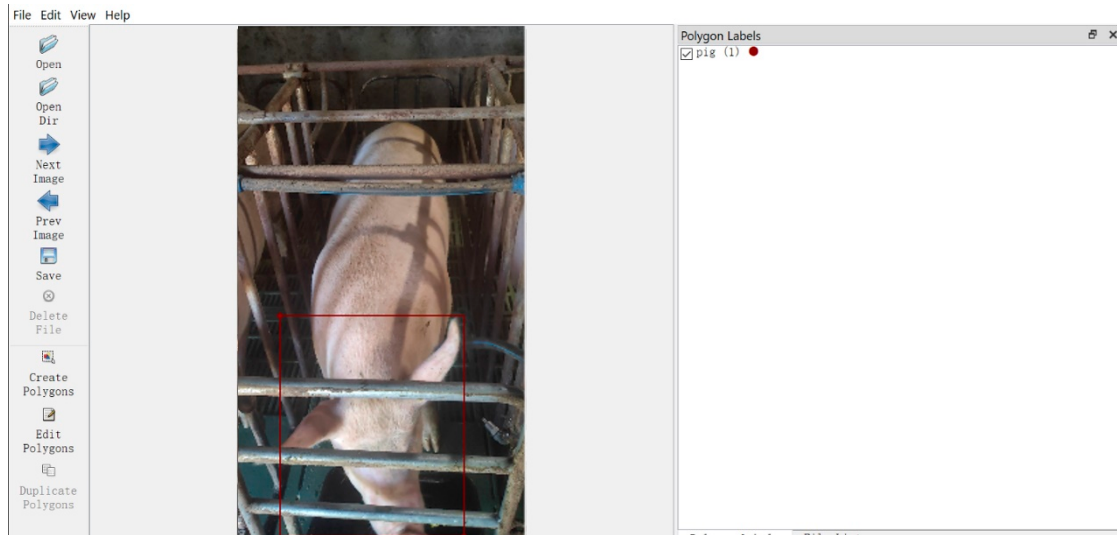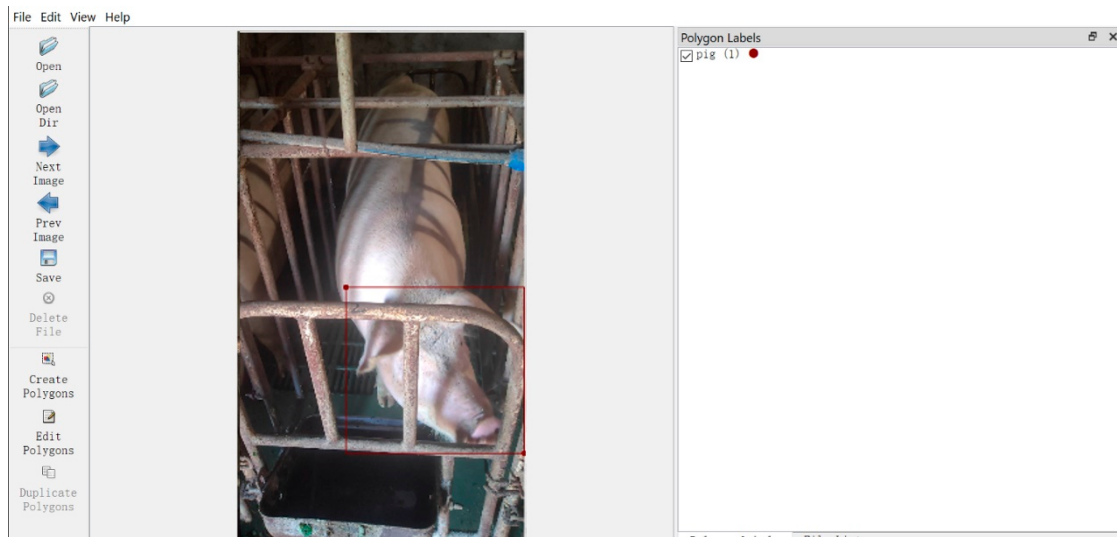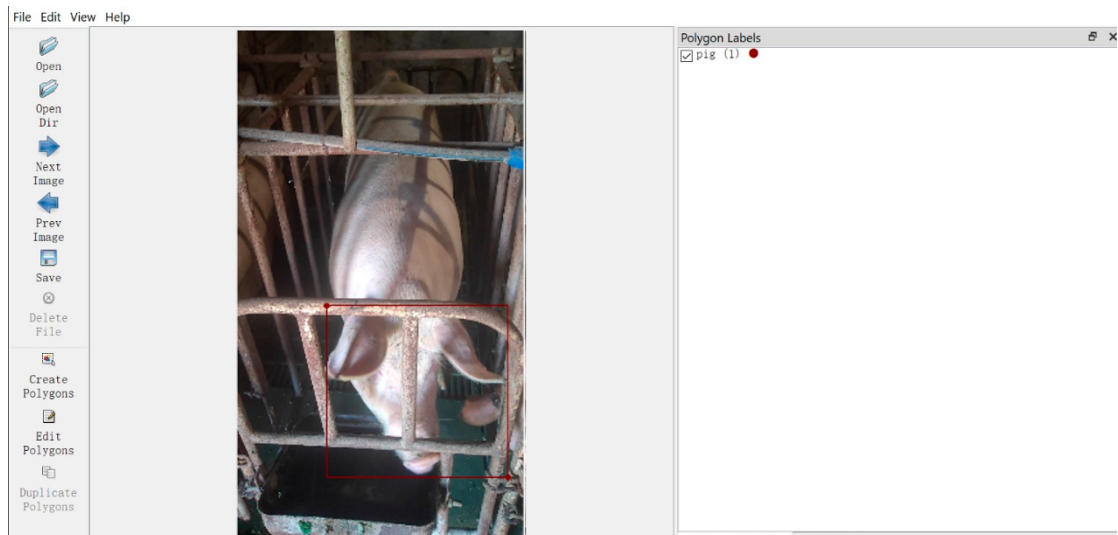

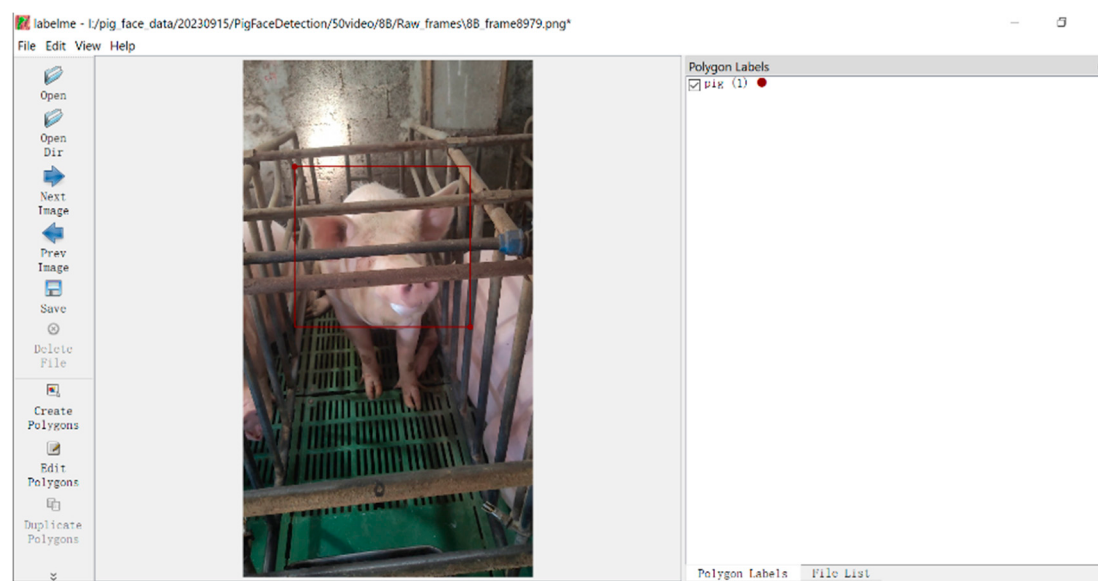

**Figure S1.** The visualization of pig face annotation using LabelMe Tool.

Supplement: Supplementary file 1 [file animals-15-00691-s001.zip › animals-3365317-supplementary.pdf]
